# A Tripartite Neurocognitive Model of Internet Gaming Disorder

**DOI:** 10.3389/fpsyt.2017.00285

**Published:** 2017-12-14

**Authors:** Lei Wei, Shuyue Zhang, Ofir Turel, Antoine Bechara, Qinghua He

**Affiliations:** ^1^Faculty of Psychology, Southwest University, Chongqing, China; ^2^Department of Psychology, Guangxi University, Guangxi, China; ^3^College of Business and Economics, California State University, Fullerton, Fullerton, CA, United States; ^4^Department of Psychology, University of Southern California, Los Angeles, CA, United States; ^5^Key Laboratory of Mental Health, Institute of Psychology, Chinese Academy of Sciences, Beijing, China; ^6^Southwest University Branch, Collaborative Innovation Center of Assessment toward Basic Education Quality at Beijing Normal University, Chongqing, China; ^7^Chongqing Collaborative Innovation Center for Brain Science, Chongqing, China

**Keywords:** Internet gaming disorder, insula, decision-making, fMRI, striatum

## Abstract

Playing Internet games has emerged as a growing in prevalence leisure activity. In some cases, excess gaming can lead to addiction-like symptoms and aversive outcomes that may be seen by some as manifestations of a behavioral addiction. Even though agreement regarding the pathologizing of excessive video gaming is not yet achieved and perhaps because the field requires more research, many works have examined the antecedents and outcomes of what is termed internet gaming disorder (IGD). In this article, we aim at summarizing perspectives and findings related to the neurocognitive processes that may underlie IGD and map such findings onto the triadic-system that governs behavior and decision-making, the deficits in which have been shown to be associated with many addictive disorders. This tripartite system model includes the following three brain systems: (1) the impulsive system, which often mediates fast, automatic, unconscious, and habitual behaviors; (2) the reflective system, which mediates deliberating, planning, predicting future outcomes of selected behaviors, and exerting inhibitory control; and (3) the interoceptive awareness system, which generates a state of craving through the translation of somatic signals into a subjective state of drive. We suggest that IGD formation and maintenance can be associated with (1) a hyperactive “impulsive” system; (2) a hypoactive “reflective” system, as exacerbated by (3) an interoceptive awareness system that potentiates the activity of the impulsive system, and/or hijacks the goal-driven cognitive resources needed for the normal operation of the reflective system. Based on this review, we propose ways to improve the therapy and treatment of IGD and reduce the risk of relapse among recovering IGD populations.

## Introduction

The Internet offers a large variety of video games, including First Person or Ego-Shooters (FPS), Massively Multiplayer Online Role Playing Games (MMORPG), Multiplayer Online Battle Arena (MOBA) games, and hybrid forms of online games, such as Overwatch, which include the elements of both MOBA and FPS. MMORPG is the most popular game type among young adults and has been the focus of many IGD studies (1). Regardless of the nature and type of the game, videogames are possibly addictive since they provide strong rewards that are difficult to resist, and which are largely encouraged by videogame developers in order to ensure that gamers keep on using their games (2). For example, they serve various functional needs of users, such as need for escapism, socialization achievement, and mastery, and are hence appealing to many young adults (3).

Research has shown that given such psychological benefits that stem from the needs served by videogames and the inability of some people to regulate their reward seeking behaviors, some players can present addiction-like symptoms in relation to videogames and that these symptoms can produce a range of aversive effects, on children (2, 4), young-adults (5, 6), and organizational employees (7–9). The concept of internet gaming disorder (IGD) has been suggested as a way to encapsulate such phenomenology and symptoms. IGD is a behavioral addiction on the spectrum of Internet addiction. It can be defined as persistent and recurrent use of the Internet to engage in games, often with other players, leading to clinically significant impairment or distress in a 12-month period (10, 11). Many studies have used adaptations or derivatives of this definition, though there is still a great deal of confusion regarding the boundaries of IGD and its measurement (12). The multiplicity of conceptualizations and measures may contribute to the different prevalence rates estimated in different studies; ranging from 0.1% to over 50% (13).

In 2013, the newly updated version of the Diagnosis and Statistical Manual for Mental Disorders (DSM-5) included IGD in its appendix and suggested nine criteria for characterizing this disorder (10, 11). These criteria are:

- preoccupation with Internet games
- withdrawal symptoms of irritability, anxiety, or sadness
- development of tolerance
- unsuccessful attempts to control the behavior
- loss of interest in other activities
- continued excessive use despite knowledge of psychosocial problems
- deceiving others regarding the amount of time spent gaming
- use of this behavior to escape or relieve a negative mood
- jeopardizing/losing a significant relationship/job/educational opportunity.

These criteria have been traditionally associated with substance-related addiction (14). Subjects should respond with yes/no to questions like “Do you spend a lot of time thinking about games even when you are not playing, or planning when you can play next?”; there is a proposed cut-off point of five criteria in DSM-5 (15). Nevertheless, proposing such criteria and cutoffs have raised a multitude of concerns regarding their ambiguity, reliance on addiction models from other domains, and reliance on prior research, which in many cases used non-clinical samples (12). Hence, many conclude that moving forward we need to conduct more research on IGD and/or better synthesize prior studies (16). Here, we venture to provide a synthesis of prior research on IGD, using a very specific angle, a neuro-cognitive one.

On the basis of recent neuro-cognitive models of addiction (17–20), and possible similarities between IGD and other addictions (13, 21–24), we suggest that the neural substrates involved in IGD development and maintenance can include the key brain systems that govern behavior and decision-making. Deficits in such systems have been shown to be associated with a broad range of addictions, including behavioral ones (17). Adapting this view, we contend that IGD may be associated with an imbalance between several inter-connected neural systems: (1) an hyperactive “impulsive” system, which is fast, automatic, and unconscious; it promotes automatic and habitual actions; (2) a hypoactive “reflective” system, which is slow and deliberative, forecasts the future consequences of a behavior and exerts inhibitory control; and (3) the interoceptive awareness system, which translates bottom-up somatic signals into a subjective state of craving, which in turn potentiates the activity of the impulsive system, and/or hijacks the goal-driven cognitive resources needed for the normal operation of the reflective system (17). In this article, we describe the connection between these three neural systems and IGD and evidence that supports this tripartite model. We use this description for pointing to potential interventions and directions for future studies.

## Addictive Properties of Internet Gaming

Addiction forms through a sensitization process (25) that changes behaviors from impulsive to compulsive. Similar to other addictive disorders that focus on behaviors (e.g., gambling), IGD cases develop an addictive state without substance intake. This can happen given the rewarding and immersive properties of videogames (26, 27) as well as their ability to address a broad spectrum of human functional needs (3). These include: relationship building, escapism, need for achievement, and mastering the game mechanics. Such motivations increase playtime and desire to play more (3), which in turn sensitizes the brain reward system (28, 29) and can lead to addiction symptoms in vulnerable populations (30).

Not all gamers will present addiction-like symptoms and meet IGD criteria, even if they play for extended periods of time (1). Research has indicates that personality traits such as avoidant traits, schizoid personality, diminished self-control, narcissism, and low self-esteem are significantly related to IGD (31). Hence, people with such traits may be more prone than others to present IGD. In addition, social-environmental factors such as pressure from school (32), which tends to be high especially in East Asia, may propel a higher prevalence rate of IGD cases in Asian countries (33, 34). Males seem to present higher IGD rates compared to women (35); and this changes when the focus is not just on games, but more broadly on Internet use (36). In the absence of prevention and harm reduction strategies that parents and educators can follow, young adults are more prone than others to lose control over online gaming (3).

Here, without discounting the importance of the many addictive features of video games, we emphasize two largely overlooked properties that many videogames have and can drive addictive behavior, if a person has deficits in the brain systems that govern decision-making:

(1) Providing a freedom space for players
  A virtual environment means that gamers can fulfill their desires that could not be met in real life and be, at least temporarily, other people with better qualities [see, for example, the notion of False Online Self in Ref. (37)]. These attributes can be highly rewarding, and present a possible reason for why game players persist in online gaming despite aversive outcomes (38). For instance, during such games, the role acted by a player could easily destroy and damage others in the virtual world and have a strong dominant personality, which may differ from the true-self of the gamer. The game space can be appealing also because it allows levels of violence that are often not afforded in real world. Many Internet games contain elements of violence; this feature may enhance interest in games and make them more rewarding, especially for young adults (39).
  In addition to violence features, Internet games also provide an environment to fulfill gamers’ desire to build an association, challenge one’s abilities, and command others (40, 41). In other words, the virtual world provides a place to escape stress from real life and one’s emotional state can be improved by playing online (3). Moreover, many Internet games allow players to pay in order to enhance the ability of the avatar representing them [in-game purchases, see, for example, Ref. (42)]. This process allows fast and easy enhancement compared to real-life attempts to enhance one’s image and persona (41). Thus, vulnerable individuals can get sucked into the virtual world and avoid the real world (43). In sum, the virtual world includes many elements that help game players fulfill voids in their real life and provide enjoyable shortcuts for achieving aspirations in a simulation world. This process brings is psychologically rewarding, sometimes more than real life. It can hence motivate consumptions that over time may translate into compulsion.
(2) Anonymity
  Anonymity has traditionally been conceived as the inability of others to identify an individual (44). Anonymity is common in many video games in which users use pseudonyms to describe themselves. This gives Internet game players a sense of security (false or not), which makes the virtual environment very appealing. In such environments, people can present abnormal behaviors and be free of direct judgment; for example, vulnerable individuals can show antisocial behaviors in online games (45). These antisocial behaviors may be linked to a loss of inhibition control (46). As such, the perceived-to-be safe environment afforded by anonymity features allows addicted users to engage in antisocial behaviors, which are aligned with their deficits in self-control abilities. When one’s true identity is not revealed, anti-social gamers do not need to take responsibility for their in-game behavior, and suspend their enjoyment in the virtual environment (47). This reduced need for self-inhibition is also very appealing, can generate strong psychological rewards, and ultimately, in vulnerable users, lead to transition from habitual gaming to compulsive gaming.

## IGD and the Impulsive Brain System (System 1)

In the course of addiction, the sensitivity to cues related to the addictive substance or behavior is progressively increased, and responses become more automatic after continuous exposure to addiction stimuli (48). This process could easily shift goal-directed behaviors to compulsive behavior, in which the action becomes independent of the current value of the goal, and result with impulsive behavior (49). Previous research indicates that impulsivity is associated with increased novelty seeking and poor decision-making and can lead to negative consequence such as monetary losses or social failures; thus, it underlies the development and maintenance of state compulsivity (50).

Recent studies found that the striatal-cortical system is a central one for acting prematurely without foresight (51). This system includes the striatum (dopaminergic systems) and the amygdala, which are key structures that form the impulsive system, and mediate reward seeking and compulsion, through sensitization (17). Accordingly, the amygdala has been repeatedly reported to be involved in risk-taking behavior; lower density of gray matter in the amygdala has been found in many substance addiction cases (52, 53) and may be perceived as indicative of making the amygdala-striatal system more efficient (28, 29).

Research has also pointed to the role of the amygdala-striatal system in IGD development and maintenance. The structures of the impulsive system have changed during the transition from goal-directed to compulsive behaviors (54). For instance, excessive play of Internet games was associated with specific aspects of synaptic structure plasticity in both striatal regions. A positron emission tomography study found that, after prolonged Internet use, the level of dopamine D2 receptor and transporters availability in subdivisions of the striatum has been reduced compared with controls (55, 56). Voxel-based morphometry research suggested that frequent Internet game playing is associated with higher volumes in the left striatal and right caudate compared with infrequent game players (57, 58), but the bilateral amygdala had a lower gray matter density in IGD cases compared to controls (59). Moreover, through the repetition of online gaming experience and exposure to gaming-related information, players learn to associate gaming with reward, and progressively become hypersensitive to gaming-related cues (60). This process can establish linkage between gaming-related cues and positive mood, which can increase dopaminergic activity and dopamine levels (61).

Moreover, a person who presents IGD symptoms can become hypersensitive to gaming-related cues; that is, develop attentional bias toward game-related cues (62), which can manifest in issues such as time distortion (63). Human behavior is determined by two aspects of cognition, implicit cognition, which includes memory association and situational circumstance, and explicit cognition, which includes cognitions amenable to introspection and deliberate decision-making (64). According to the implicit association test, which is used to asses implicit associations, players with IGD have a positive motivational implicit response to screenshots of games (65), including in cases of first-person shooter and racing games (66). These findings indicate a strong association between implicit cognition and uncontrolled gaming behavior. Implicit cognition not only represents an automatic appetitive response to a specific substance but can also impact specific behaviors, such as playing online videogames. Because implicit cognitions play an important role in addictive behavior through the generation of automatic approach tendencies, and these cognitions are often mediated *via* the amygdala–striatal system, the modulation of this system can be associated with addictive behaviors (67, 68), including the presumed-to-be addictive and problematic use of technologies (6, 20, 28, 29, 69, 70, 71).

fMRI studies also point to differences between brain activity of the impulsive system of presumed IGD and non-IGD cases. Both arterial spin-labeling perfusion and functional magnetic resonance imaging found differences during resting state: IGD subjects showed significantly higher global cerebral blood flow in the left parahippocampal and amygdala (72) and revealed reduced functional connectivity with fronto-striatal circuits (73, 74). Studies using the cue-reactivity paradigm indicated higher activation of the striatum among IGD subjects, compared to controls (26, 75). They further suggested functional differences between dorsal and ventral striatal subdivisions. After presenting game-related stimuli and neutral stimuli, the left ventral striatum activity of IGD cases showed negative correlation with cue-induced craving, but dorsal striatal activation was positively associated with duration of IGD. Hence, the transition from ventral to dorsal striatal processing of addiction-related cues may occur among IGD individuals (76).

Overall, continuously playing online can build a strong association between reward and behavior schema, and this association is mainly mediated by the amygdala-striatal system (77); impairment of this system can be associated with addictions in general (17) and specifically IGD (26, 27). The impairment of the impulsive system may be similar across addictions and problematic behaviors (78). Hence, it is not surprising to see structural, functional and connectivity abnormalities in this system in presumed-to-be IGD cases.

## IGD and the Reflective Brain System (System 2)

The reflective system can be conceived as a controller of the motivation toward addiction related reward and the impulsive behavior that is produced by impulsive system. The reflective system forecasts the result of current behavior and allows more flexible pursuit of long-term goals. This system consists of two sets of neural systems: a “cool” system (elicited by relatively abstract, decontextualized problems, and refers to basic working memory operations, inhibition of prepotent impulsions, and mental set shifting) and a “hot” system (involved in triggering somatic states from memory, knowledge, cognition, and activates numerous affective/emotional (somatic) response that conflict with each other) (79).

Studies indicated that the cool executive functions are mainly dependent on the lateral inferior and dorsolateral prefrontal cortices, and the anterior cingulate cortex, and that they are involved in several kinds of psychological reaction, such as shifting between multiple tasks and the updating or maintaining of working memory (79). In contrast to the cool executive functions, the orbitofrontal cortex (OFC) and ventromedial prefrontal cortex (VMPFC) form the main structure of hot executive functions. These are involved in the interaction between affective/emotional responses and somatic states that produce overall positive or negative signals related to behavioral choices (79).

### IGD and Hot Executive Function

The disruption of hot executive function in addiction has been initially demonstrated in clinical research of patient populations with damage in frontal lobe regions. These studies showed that hot executive function disruption delineates similar result to those obtained in cases of impairment to the frontal cortex (80, 81). The Iowa Gambling Task (IGT) has been typically applied in such addiction studies, to examine decision-making abilities under ambiguity (82). This paradigm was introduced as a tool to measure “risk-anticipation,” which involves probabilistic learning *via* monetary rewards and punishments (83). Results of IGT studies demonstrated a reduced decision-making ability compared to controls during the task; they also show that presumed IGD cases made more disadvantageous decisions and performed worse than healthy controls (40, 84, 85). Excessive game playing that results in addiction-like symptoms, therefore, may be associated with deficient ability to integrate previous emotional/affective experiences of rewards or punishments, to motivate and engage in inhibition as well as to trigger somatic responses.

According to the somatic marker hypothesis, somatic response is multidimensional and the emotional experience caused by the reward or punishment under a decision-making situation, would change with the somatic state (86). Adapting this view, one can argue that IGD may be associated with impaired reward and punishment expectation and processing functions. Support for this view has been given in a study on the underlying neural mechanisms of disadvantageous risky decision-making in IGD cases. During the Balloon Analog Risk Task (BART), a significant interaction effect between risk level and activation of the bilateral ventral medial prefrontal cortex (PFC) has been shown (87). Another study, which used a modified delay-discounting task, also suggested that IGD cases prefer the probabilistic or risky options; it also showed that there is a positive correlation between activation of inferior frontal gyrus and probability discounting rates (88).

In contrast, evidence from First Person or Ego-Shooters players suggests that excessive videogame playing may enhance the performance on an IGT compared to controls (89), while experience with First Person or Ego-Shooters games was positively correlated with impulsivity, and experience with strategy games was negatively correlated with impulsivity (85). One reasonable interpretation is that First Person or Ego-Shooters games include many violent elements, which could arouse the impulsive system (90, 91). The most popular type of game, Multiplayer Online Role Playing game, can also contain violent scenes (92). Indeed, studies suggest relation between IGD and aggression (91), which may manifest from deficits in the hot inhibition/control brain system. In other words, after prolonged exposure to violent games, IGD cases may develop higher aggression than healthy subjects, which would promote their risk-taking intentions and behaviors (93).

Several studies have also reported that structural impairment in the orbital frontal cortex in IGD cases. These impairments include abnormal glucose metabolism, abnormal of cortical thickness, and white matter fiber consistency (94–96). Moreover, compared to the neutral pictures, gaming pictures activated the OFC, right nucleus accumbens and bilateral Anterior Cingulate Cortex (ACC) (26). These results demonstrate that the orbital frontal cortex is involved in the modulation of reactive aggression; simply put the orbital frontal cortex fails to “inhibit” reactive aggression in response to social cues present in the environment (97).

Distinguishing it from other addictive substances and behaviors, video gaming provides different kinds of scenes and environments that can constantly stimulate use, rewards, violence and arousal. This emotional aspect that is apparent especially in violent games can lead to mood changes and disrupt the integration of emotional and cognitive inputs in the orbital frontal cortex (98). This process can also increase impulsivity, tendency for risk-taking and ignoring negative effects while seeking further rewards. The antisocial behavior among IGD cases suggests an association between aggression and excessive play of violent videogames (99). Overall, excessive play of online games can disrupt the hot executive system in two ways. First, the dysfunction of the ventral medial PFC impacts the value evaluation of rewards and punishments (100). Second, game-related cues arouse the mood with aggression, and this can affect the integration of emotional inputs into decision-making. The somatic state would be influenced by the aggression, and as a result, IGD cases develop impulsive tendencies as manifested in impairments to the orbital frontal cortex and the balance mediated by the orbital and ventral medial cortices is infringed upon.

### IGD and Cold Executive Function

The ability to suppress automatic and pre-potent response behaviors is critical to the prevention of addictive behaviors. Accordingly, IGD cases showed impairment of inhibition control across many studies (58, 101). Reduction in inhibition of pre-potent responses may essentially make incentive habits more powerful and increase their status to become a “default” automatic habit system (102). This happens because impaired response inhibition could lead to abnormal salience attribution toward gaming-related cues in IGD cases.

Through the paradigms of stop-signal (102) and go/no-go tasks (103), researchers could measure the ability to inhibit advantage response irrelevant to the current task or topic. Subjects were required to withhold response while a particular stop signal (stop-signal task) or stimuli occurs (go/no-go tasks). IGD cases showed impaired inhibition control while they performed relevant go/no-go tasks (such as responding faster to stimuli pictures than to neutral pictures and making more false responses than healthy subjects did) (104–107). A similar picture emerged from studies based on the stop signal task (108, 109). Considering the characteristics of online games, which include many well-designed stimuli (e.g., arousing scenes or pictures), the video-game-special go/no-go task is deemed suitable for videogame addiction research.

Results from recent brain imaging studies suggested that IGD can be associated with a disruption of brain circuits involved in motor response inhibition. Excessive gaming experience is associated with increased gray matter in the right hippocampal formation, dorsolateral PFC, and bilateral cerebellum (110, 111). Resting state studies find decreased functional connectivity in the PFC—striatal circuit in IGD cases (112). Using the go/no-go task, a significantly hyperactive left superior medial frontal and right anterior cingulate cortex during no-go trials was found (105). Using gaming-related picture as cues, healthy controls increased brain activation in the right dorsolateral PFC in comparison with the IGD cases (113). Moreover, 6 months therapy of Bupropion, which is used in the treatment of substance disorders, reduced relevant activations in response to the game-related cues, in IGD cases (114). These results point to possible abnormalities in presumed IGD cases in terms of cold executive function. They show that prolonged playing, sensitizes the impulsive brain systems and when coupled with deficits in executive control (115), it can lead to difficulty to inhibit prepotent game cues and to the emergence of addiction-like symptoms (116).

## The Interoceptive Processes (System 3)

Previous research has suggested that an interoceptive system can modulate the balance between the impulsive and reflective systems, and that the exacerbated imbalance can help maintaining addictions (20). The main function of interoceptive processes is sensing psychological and physical imbalances and mediating response signals in the form of disgust, craving, urge, etc. as a means to signal the need to restore homeostasis. In the case of addiction, this system mediates anticipation for rewards by translating somatic sensory signals into one’s subjectively experience of a desire to engage in the behavior (117–119). This process mainly depends on the structure of bilateral insular cortex (120).

### The Insula and IGD

Studies have shown that the insular cortex plays an important role in substance dependence and seeking (121, 122). This happens because the translating of somatic signals into subjective experience of craving increases sensitivity toward addiction-related cues and can reduce inhibition resources availability (118, 120). Indeed, the activation of the insular cortex has been implicated in a wide range of conditions and behaviors, such as anticipating the future results about monetary gains (123) or losses (124). Accordingly, the thickness of insular cortex was negatively associated with cigarette exposure response (125), while damage to the insular cortex could disrupt cigarette smoking; smokers with damage to insula quit smoking easily and show a higher rate of cessation from smoking which is nearly 100 times more than this of smokers without damage to insula (126).

The formation of interoceptive system representation through insular cortex activation is crucial for decision-making regarding prepotent cues (118). Considering the position of the insular cortex in the brain, it can be seen as a bridge between ventromedial and OFC and the impulsive system regions. As such, the insula has been suggested to act as a connector that translates somatic signals and triggers bodily states (118). The co-activation pattern between the insula and the ventromedial frontal cortex has been revealed during the process of generating somatic markers that involved reference judgments (127). By working in tandem with the vmPFC, the insula could map the relationship between external objects and internal somatic sensory states, and invoke bodily states.

Recent studies also suggest that the insula plays an important role in IGD. They revealed decreased functional connectivity between the insula and the motor/executive cortices (such as dlPFC, OFC, cingulated cortex) in IGD cases (128, 129). This finding revealed weaken connections between the insula and the reflective system among IGD individuals, which might explain loss of control in such cases. As such, in IGD cases the insula can be presumed to have abnormal abilities to communicate with the executive system. While exposed to game-related pictures, the insula has been activated and the activation was positively correlated with self-reported gaming urge stimulated by the pictures (26, 27). This may reveal that the insula is related to the relationship between rewarding cues and the craving level one subjectively experience.

Evidence from co-activation research also suggested strong association between the insula and the impulsive and reflective systems; in the presence of game-related cues, co-activation patterns in orbital frontal cortex, insula, anterior cingulate cortex, and dorsolateral cortex have been observed (26). These findings provide further support to the hypothesis that the key role of the insula is to serve as a hub mediating craving production through communication with impulsive and reflective brain systems.

The insula also plays an important role in the development and maintenance of addiction; it integrates the interoceptive effects of addictive substances or behaviors into conscious awareness, memory, or executive functions (130). In support of this view, research has indicated that deficit in response inhibition is pronounced during periods of heightened motivational state of drug intake (131) or drinking alcohol (132). These deficits are triggered by the high subjective state during the stage of abstinence while the affective stimuli related to the addiction substance consume enormous attentional resources and result in the disruption of inhibitory control. Under such overload of attentional resources, the attraction caused by the stimuli may encourage relapse and make it difficult to overcome tempting addictive behaviors (131, 132). In other words, insula-mediated interoceptive representations have the capacity to “hijack” the cognitive resources necessary for exerting inhibitory control to resist the temptation to smoke, use drugs, or use social media impulsively (20) by disabling activity of the prefrontal (control/reflective) system. The anterior insula has bidirectional connections to the amygdala, ventral striatum, and OFC. The insula integrates the interoceptive state into conscious feelings and into decision-making processes that involve certain risks and rewards; it presents decreased cortical thickness in IGD cases (94, 133). This structural abnormality of the interoceptive system may also hamper self-awareness, which could take the form of failure to recognize an illness (134). Young adults with high levels of IGD often also present depression, anxiety, aggression, or social phobias symptoms (135). Such symptoms may also be associated with dysfunction of the translation of interoceptive signals emerging from somatic and emotional states (136). Moreover, deprivation interoceptive signals (e.g., when one cannot play videogames even if he or she strongly desires to do do) may also hamper metacognitive abilities in addicts (137). This abnormal degree of dissociation in addicted people, between the “object” level and the “meta” level, raises the possibility that poor metacognition lead to action and decision-making monitoring and adjustment (138). Hence, when metacognitive judgment becomes exceedingly disrupted, the repetition of addictive behaviors may be heightened by an underestimation of addiction severity.

**Figure 1:**
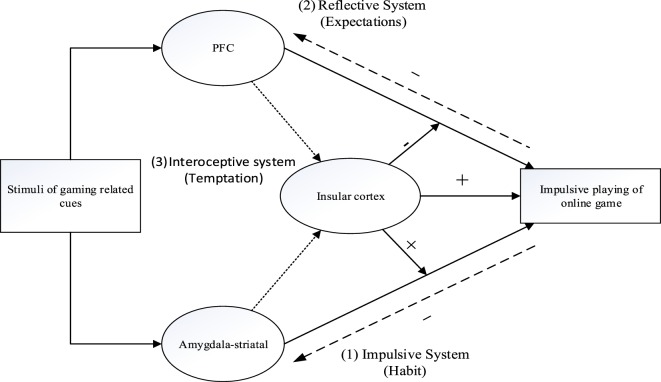
A schematic tripartite neurological model illustrating the key systems that may underlie IGD, (1) gaming related cues excite the impulsive system, which mainly depend on the amygdala and striatum, and activates cue-action links through mental associations, (2) the reflective system mainly depends on the structure of the prefrontal cortex (PFC) and inhibits the impulsions toward an Internet game, (3) the interoceptive awareness systems plays a key role in modulating the equilibrium between system 1 and system 2. Through translating interoceptive signals, the insular cortex maintains craving for an Internet game. The activity of the insular cortex increases the drive to play the Internet game and weakens the inhibition abilities regarding this action. The excessive and problematic play of online games can also invoke changes in the relevant brain regions, and by so doing exacerbate or help expressing other mental health problem.

The tripartite view that includes three systems of IGD that emerges from this review is presented in Figure 1.

## Discussion

In this article, we reviewed the neurocognitive processes that may underlie presumed IGD. This is important as many young adults (but not all) lose the ability to resist the reward and pleasure from virtual gaming worlds. That is, for some heavy gamers, an inability to resist unreal rewards emerges, despite mounting monetary, social and performance losses leading to personal, familial, financial, professional, and legal negative consequences. This loss of control that is termed IGD, we argue, may be sub-served by a tripartite network of brain systems.

Specifically, the review we provide in this paper suggests that the continuous engagement in videogame playing in IGD cases can be explained by increased automatic motivational response directed at gaming-related behaviors coupled with a lowered efficiency of impulse control and self-reflective processes, and that this imbalance may be further accentuated by abnormal interoceptive awareness processes. This tripartite view of the brain systems involved in addictive disorders (20) as applied to IGD cases here, has received support in various studies; albeit such studies have typically provided a disjointed view regarding the three involved systems. They specifically show that failure to self-control is associated with dysfunction of the impulsive and reflective brain systems (functionally and structurally) and that this dysfunction can be regulated by insular activity, the dysfunction of which can augment the imbalance between reflective and impulsive brain processes. The translation of interoceptive signals in the insula disrupted this balance by changes in somatic states that were aroused by addiction-related stimuli (videogame cues in our case). In addition, impairment in the interoceptive awareness system leads IGD cases to often ignore the negative effects of excessive playing. This increases the probability of relapse in IGD cases. Overall, online gaming provides many rewards to users and can have positive effects on many children (139). However, these same rewards can exploit brain deficits in the impulsive, reflective, and interceptive brain systems and create dysfunctions in learning, motivation, an assessment of the salience of a video game-related stimuli, to such an extent that the vulnerable individual develops addiction-like symptoms in relation to videogame playing.

Previous research has proposed several models of IGD, which are also in line with the framework we present here, but put different emphasis or ignore interoceptive awareness processes. Davis (140) argued that there are differences between generalized pathological Internet use (GIU) and specific Internet use (SIU) and suggested a cognitive behavior model to explain such differences. According to this model, maladaptive cognition of the external environment drives a series of internal responses such as negative emotions and increases the use of specific rewarding application over the Internet (e.g., online gaming, pornography). This model provides support for the assumptions in our model as both allude to the idea that maladaptive cognitions may underlie IGD; our model points to brain regions that are likely involved in developing and maintaining such cognitions.

On the basis of this research, neurocognitive models have been developed and emphasized the importance of executive function in SIU (18). These overlap with regions we discussed: the VMPFC and dorsolateral lateral PFC are suggested to be most likely involved in development and maintenance of addictive use of Internet applications. Again, this model overlaps some aspects of our model, but our model puts stronger emphasis on interoceptive awareness processes. Similarly, Dong and Potenza (141) proposed a cognitive behavior model for IGD. The model contains three key cognitive domains of IGD: motivational drive and reward-seeking, behavioral control and executive control, and decision-making as related to the long-term negative consequence of current behavioral choices. This model also emphasizes the importance of seeking motivation and the state of craving, and suggests that the state of craving may contribute to the IGD process. This is similar to our model in terms of components but does not specifically focus on the regions involved in craving generation. Similarly, a process model called Person-Affect-Cognition-Execution (I-PACE) suggests that addiction may result from increasing exposure to addiction-related cues and may involve deficits in the personal, affective, cognition, and execution domains. This model is also aligned with our neurocognitive model as personal, affective, cognition and execution domains can be mapped onto the tripartite view we present.

According to our review of neuro-cognitive studies, the dysfunction of brain structure and activations that sub-serves IGD may be similar to this in cases of substance and behavioral addictions. The impairment of the impulsive and reflective processes showed that IGD shares common mechanisms with substance addictions. They showed that prolonged excessive playing can be associated with structural and connectivity abnormalities in relevant brain regions. Importantly, such studies hint at ways through which IGD can be treated; though such approaches should be further examined in future research. First, several studies suggest the bupropion could reduce the craving and urge for video gaming (114, 142). This can be a viable treatment option, but future research should examine its efficacy given different profiles of comorbidity that are plausible in IGD cases.

Second, cognitive behavioral therapy has been most widely used for IGD treatment. It aims at moderating the impulsive processes or at boosting reflective resources such that IGD cases learn to better cope with their inability to resist gaming. For instance, after recognize the inappropriateness of their behavior, IGD cases may learn to adjust their behavioral patterns and choices (143). Such approaches should also be further studied, especially since they assume relatively intact prefrontal brain regions. This seems to be the case in mild to medium addiction levels (28, 69), but in severe IGD cases, there may be abnormalities in prefrontal regions that will not allow successful cognitive behavioral therapy. This idea merits future research.

## Author Contributions

LW, OT, AB, and QH were responsible for the study conception and design; LW and SZ wrote the first draft of the paper. SZ, OT, and QH also contributed to the writing of the paper. LW, SZ, OT, AB, and QH made the critical revision of the article. All authors gave the final approval of the article.

## Conflict of Interest Statement

The authors declare that the research was conducted in the absence of any commercial or financial relationships that could be construed as a potential conflict of interest.

## References

[1] van Rooij AJ Schoenmakers TM Vermulst AA van den Eijnden R van de Mheen D Online video game addiction: identification of addicted adolescent gamers. Addiction (2011) 106:205–12. 10.1111/j.1360-0443.2010.03104.x 20840209

[2] Turel O Romashkin A Morrison KM. Health outcomes of information system use lifestyles among adolescents: videogame addiction, sleep curtailment and cardio-metabolic deficiencies. PLoS One (2016) 11:e0154764. 10.1371/journal.pone.0154764 27149512 PMC4858285

[3] Xu Z Turel O Yuan Y Online game addiction among adolescents: motivation and prevention factors. Eur J Inform Syst (2012) 21:321–40. 10.1057/ejis.2011.56

[4] Turel O Romashkin A Morrison KM. A model linking video gaming, sleep quality, sweet drinks consumption and obesity among children and youth. Clin Obes (2017) 7:191–8. 10.1111/cob.12191 28320073

[5] Turel O Mouttapa M Donato E Preventing problematic Internet use through video-based interventions: a theoretical model and empirical test. Behav Inf Technol (2015) 34:349–62. 10.1080/0144929X.2014.936041

[6] Turel O Qahri-Saremi H Problematic use of social networking sites: antecedents and consequence from a dual-system theory perspective. J Manage Info Syst (2016) 33:1087–116. 10.1080/07421222.2016.1267529

[7] Turel O Serenko A Bontis N Family and work-related consequences of addiction to organizational pervasive technologies. Inform Manag (2011) 48:88–95. 10.1016/j.im.2011.01.004

[8] Tarafdar M Gupta A Turel O The dark side of information technology use. Info Syst J (2013) 23:269–75. 10.1111/isj.12015

[9] Tarafdar M D’Arcy J Turel O Gupta A The dark side of information technology. MIT Sloan Manage Rev (2015) 56:600–23.

[10] American Psychiatric Association. Diagnostic and Statistical Manual of Mental Disorders. Arlington: American Psychiatric Publishing (2013).

[11] American Psychiatric Association. Internet gaming disorder. 5th ed Diagnostic and Statistical Manual of Mental Disorders. Arlington, VA: American Psychiatric Publishing (2013). p. 795–8.

[12] Van Rooij AJ Kardefelt-Winther D Lost in the chaos: flawed literature should not generate new disorders: commentary on: chaos and confusion in DSM-5 diagnosis of internet gaming disorder: issues, concerns, and recommendations for clarity in the field. J Behav Addict (2017) 6:128–32. 10.1556/2006.6.2017.015 28301968 PMC5520115

[13] Petry NM Rehbein F Ko CH O’Brien CP. Internet gaming disorder in the DSM-5. Curr Psychiatry Rep (2015) 17:72. 10.1007/s11920-015-0610-0 26216590

[14] Tao R Huang X Wang J Zhang H Zhang Y Li M. Proposed diagnostic criteria for internet addiction. Addiction (2010) 105:556–64. 10.1111/j.1360-0443.2009.02828.x 20403001

[15] Petry NM Rehbein F Gentile DA Lemmens JS Rumpf HJ Mößle T An international consensus for assessing internet gaming disorder using the new DSM-5 approach. Addiction (2014) 109:1399 10.1111/add.12457 24456155

[16] Kardefelt-Winther D Heeren A Schimmenti A van Rooij A Maurage P Carras M How can we conceptualize behavioural addiction without pathologizing common behaviours? Addiction (2017) 112:1709–15. 10.1111/add.13763 28198052 PMC5557689

[17] Noël X Brevers D Bechara A. A neurocognitive approach to understanding the neurobiology of addiction. Curr Opin Neurobiol (2013) 23:632–8. 10.1016/j.conb.2013.01.018 23395462 PMC3670974

[18] Brand M Young KS Laier C Prefrontal control and Internet addiction: a theoretical model and review of neuropsychological and neuroimaging findings. Front Hum Neurosci (2014) 8:375 10.3389/fnhum.2014.00375 24904393 PMC4034340

[19] Brand M Young KS Laier C Wölfling K Potenza MN. Integrating psychological and neurobiological considerations regarding the development and maintenance of specific Internet-use disorders: an interaction of person-affect-cognition-execution (I-PACE) model. Neurosci Biobehav Rev (2016) 71:252–66. 10.1016/j.neubiorev.2016.08.033 27590829

[20] Turel O Bechara A A triadic reflective-impulsive-interoceptive awareness model of general and impulsive information system use: behavioral tests of neuro-cognitive theory. Front Psychol (2016) 7:601 10.3389/fpsyg.2016.00601 27199834 PMC4845517

[21] Rehbein F Kliem S Baier D Mossle T Petry NM. Prevalence of internet gaming disorder in German adolescents: diagnostic contribution of the nine DSM-5 criteria in a state-wide representative sample. Addiction (2015) 110:842–51. 10.1111/add.12849 25598040

[22] Kiraly O Sleczka P Pontes HM Urban R Griffiths MD Demetrovics Z. Validation of the ten-item internet gaming disorder test (IGDT-10) and evaluation of the nine DSM-5 internet gaming disorder criteria. Addict Behav (2017) 64:253–60. 10.1016/j.addbeh.2015.11.005 26632194

[23] Koo HJ Han DH Park SY Kwon JH. The structured clinical interview for DSM-5 Internet gaming disorder: development and validation for diagnosing IGD in adolescents. Psychiatry Invest (2017) 14:21–9. 10.4306/pi.2017.14.1.21 28096871 PMC5240456

[24] Yao YW Potenza MN Zhang JT Internet gaming disorder within the DSM-5 framework and with an eye toward ICD-11. Am J Psychiatry (2017) 174:486–486. 10.1176/appi.ajp.2017.16121346 28457151

[25] Robinson TE Berridge KC Incentive-sensitization and addiction. Addiction (2001) 96:103–14. 10.1046/j.1360-0443.2001.9611038.x 11177523

[26] Ko C-H Liu G-C Hsiao S Yen J-Y Yang M-J Lin W-C Brain activities associated with gaming urge of online gaming addiction. J Psychiatr Res (2009) 43:739–47. 10.1016/j.jpsychires.2008.09.012 18996542

[27] Ko CH Liu GC Yen JY Chen CY Yen CF Chen CS. Brain correlates of craving for online gaming under cue exposure in subjects with Internet gaming addiction and in remitted subjects. Addict Biol (2013) 18:559–69. 10.1111/j.1369-1600.2011.00405.x 22026537

[28] He Q Turel O Bechara A Brain anatomy alterations associated with social networking site (SNS) addiction. Sci Rep (2017) 7:1–8. 10.1038/srep45064 28332625 PMC5362930

[29] He Q Turel O Brevers D Bechara A Excess social media use in normal populations is associated with amygdala-striatal but not with prefrontal morphology. Psychiatry Res Neuroimag (2017) 269:31–5. 10.1016/j.pscychresns.2017.09.003 28918269

[30] Turel O Serenko A The benefits and dangers of enjoyment with social networking websites. Eur J Inform Syst (2012) 21:512–28. 10.1057/ejis.2012.1

[31] Kuss DJ Griffiths MD Internet gaming addiction: a systematic review of empirical research. Int J Mental Health Addict (2012) 10:278–96. 10.1007/s11469-011-9318-5

[32] Niemz K Griffiths M Banyard P. Prevalence of pathological Internet use among university students and correlations with self-esteem, the General Health Questionnaire (GHQ), and disinhibition. Cyberpsychol Behav (2005) 8:562. 10.1089/cpb.2005.8.562 16332167

[33] Hur MH. Demographic, habitual, and socioeconomic determinants of Internet addiction disorder: an empirical study of Korean teenagers. Cyberpsychol Behav (2006) 9:514. 10.1089/cpb.2006.9.514 17034317

[34] Choo H Gentile DA Sim T Li D Khoo A Liau AK. Pathological video-gaming among Singaporean youth. Ann Acad Med Singapore (2010) 39:822–9. 21165520

[35] Ko CH Yen JY Chen CC Chen SH Yen CF. Gender differences and related factors affecting online gaming addiction among Taiwanese adolescents. J Nerv Mental Dis (2005) 193:273. 10.1097/01.nmd.0000158373.85150.57 15805824

[36] Yen JY Yen CF Chen CS Tang TC Ko CH. The association between adult ADHD symptoms and internet addiction among college students: the gender difference. Cyberpsychol Behav (2009) 12:187. 10.1089/cpb.2008.0113 19072077

[37] Gil-Or O Levi-Belz Y Turel O The “Facebook-self”: characteristics and psychological predictors of false self-presentation on Facebook. Front Psychol (2015) 6:99 10.3389/fpsyg.2015.00099 25741299 PMC4330900

[38] Whang LS-M Chang G Lifestyles of virtual world residents: living in the on-line game “Lineage”. Cyberpsychol Behav (2004) 7:592–600. 10.1089/cpb.2004.7.592 15667054

[39] Madran HAD Cakilci EF The relationship between agression and online video game addiction: a study on massively multiplayer online video game players. Anadolu Psikiyatri Dergisi Anatolian J Psychiatry (2014) 15:99–107. 10.5455/apd.39828

[40] Pawlikowski M Brand M. Excessive Internet gaming and decision making: do excessive World of Warcraft players have problems in decision making under risky conditions? Psychiatry Res (2011) 188:428–33. 10.1016/j.psychres.2011.05.017 21641048

[41] Billieux J Van der Linden M Achab S Khazaal Y Paraskevopoulos L Zullino D Why do you play World of Warcraft? An in-depth exploration of self-reported motivations to play online and in-game behaviours in the virtual world of Azeroth. Comput Hum Behav (2013) 29:103–9. 10.1016/j.chb.2012.07.021

[42] Hamari J Alha K Jarvela S Kivikangas JM Koivisto J Paavilainen J Why do players buy in-game content? An empirical study on concrete purchase motivations. Comput Hum Behav (2017) 68:538–46. 10.1016/j.chb.2016.11.045

[43] Yee N. Motivations for play in online games. Cyberpsychol Behav (2006) 9:772–5. 10.1089/cpb.2006.9.772 17201605

[44] Christopherson KM The positive and negative implications of anonymity in Internet social interactions: “on the internet, nobody knows you’re a dog”. Comput Hum Behav (2007) 23:3038–56. 10.1016/j.chb.2006.09.001

[45] Ma HK. Internet addiction and antisocial internet behavior of adolescents. Sci World J (2011) 11:2187–96. 10.1100/2011/308631 22125466 PMC3217592

[46] Catalano RF Hawkins JD A theory of antisocial behavior. In: Hawkins JD, editor. Delinquency and Crime: Current Theories. New York: Cambridge University Press (1996). p. 149–97.

[47] Bowman ND Schultheiss D Schumann C “I’m attached, and I’m a good guy/gal!”: how character attachment influences pro- and anti-social motivations to play massively multiplayer online role-playing games. Cyberpsychol Behav Soc Network (2012) 15:169 10.1089/cyber.2011.0311 22339552

[48] Everitt BJ Robbins TW. Neural systems of reinforcement for drug addiction: from actions to habits to compulsion. Nat Neurosci (2005) 8:1481–9. 10.1038/nn1579 16251991

[49] Dickinson A Balleine B Watt A Gonzalez F Boakes RA Motivational control after extended instrumental training. Learn Behav (1995) 23:197–206. 10.3758/BF03199935

[50] Chambers RA Taylor JR Potenza MN. Developmental neurocircuitry of motivation in adolescence: a critical period of addiction vulnerability. Am J Psychiatry (2003) 160:1041–52. 10.1176/appi.ajp.160.6.1041 12777258 PMC2919168

[51] Dalley JW Everitt BJ Robbins TW. Impulsivity, compulsivity, and top-down cognitive control. Neuron (2011) 69:680–94. 10.1016/j.neuron.2011.01.020 21338879

[52] Connolly CG Bell RP Foxe JJ Garavan H. Dissociated grey matter changes with prolonged addiction and extended abstinence in cocaine users. PLoS One (2013) 8:e59645. 10.1371/journal.pone.0059645 23527239 PMC3601087

[53] Gilman JM Kuster JK Lee S Lee MJ Kim BW Makris N Cannabis use is quantitatively associated with nucleus accumbens and amygdala abnormalities in young adult recreational users. J Neurosci (2014) 34:5529–38. 10.1523/JNEUROSCI.4745-13.2014 24741043 PMC3988409

[54] Grueter BA Rothwell PE Malenka RC. Integrating synaptic plasticity and striatal circuit function in addiction. Curr Opin Neurobiol (2012) 22:545–51. 10.1016/j.conb.2011.09.009 22000687 PMC3276730

[55] Kim SH Baik S-H Park CS Kim SJ Choi SW Kim SE. Reduced striatal dopamine D2 receptors in people with Internet addiction. Neuroreport (2011) 22:407–11. 10.1097/WNR.0b013e328346e16e 21499141

[56] Hou H Jia S Hu S Fan R Sun W Sun T Reduced striatal dopamine transporters in people with internet addiction disorder. Biomed Res Int (2012) 2012:854524 10.1155/2012/854524 PMC3312312 22505818

[57] Kühn S Romanowski A Schilling C Lorenz R Mörsen C Seiferth N The neural basis of video gaming. Trans Psychiatry (2011) 1:e53 10.1038/tp.2011.53 PMC3309473 22833208

[58] Cai C Yuan K Yin J Feng D Bi Y Li Y Striatum morphometry is associated with cognitive control deficits and symptom severity in internet gaming disorder. Brain Imaging Behav (2016) 10:12–20. 10.1007/s11682-015-9358-8 25720356

[59] Ko C-H Hsieh T-J Wang P-W Lin W-C Yen C-F Chen C-S Altered gray matter density and disrupted functional connectivity of the amygdala in adults with Internet gaming disorder. Prog Neuropsychopharmacol Biol Psychiatry (2015) 57:185–92. 10.1016/j.pnpbp.2014.11.003 25448779

[60] Turel O Serenko A Giles P Integrating technology addiction and use: an empirical investigation of online auction sites. MIS Q (2011) 35:1043–61. 10.2307/41409972

[61] Han DH Lee YS Yang KC Kim EY Lyoo IK Renshaw PF. Dopamine genes and reward dependence in adolescents with excessive internet video game play. J Addict Med (2007) 1:133–8. 10.1097/ADM.0b013e31811f465f 21768948

[62] Lorenz RC Krüger JK Neumann B Schott BH Kaufmann C Heinz A Cue reactivity and its inhibition in pathological computer game players. Addict Biol (2013) 18:134–46. 10.1111/j.1369-1600.2012.00491.x 22970898

[63] Turel O Brevers D Bechara A. Time distortion when users at-risk for social media addiction engage in non-social media tasks. J Psychiatr Res (2018) 97:84–8. 10.1016/j.jpsychires.2017.11.014 29220826

[64] McCarthy DM Thompsen DM. Implicit and explicit measures of alcohol and smoking cognitions. Psychol Addict Behav (2006) 20:436. 10.1037/0893-164X.20.4.436 17176178

[65] Yen J-Y Yen C-F Chen C-S Tang T-C Huang T-H Ko C-H. Cue-induced positive motivational implicit response in young adults with Internet gaming addiction. Psychiatry Res (2011) 190:282–6. 10.1016/j.psychres.2011.07.003 21820184

[66] Klimmt C Hefner D Vorderer P Roth C Blake C Identification with video game characters as automatic shift of self-perceptions. Media Psychol (2010) 13:323–38. 10.1080/15213269.2010.524911

[67] Ames SL Grenard JL Stacy AW Xiao L He Q Wong SW Functional imaging of implicit marijuana associations during performance on an implicit association test (IAT). Behav Brain Res (2013) 256:494–502. 10.1016/j.bbr.2013.09.013 24029699 PMC3836671

[68] Ames SL Grenard JL He Q Stacy AW Wong SW Xiao L Functional imaging of an alcohol-implicit association test (IAT). Addict Biol (2014) 19:467–81. 10.1111/adb.12071 23822813 PMC4116809

[69] Turel O He Q Xue G Xiao L Bechara A Examination of neural systems sub-serving Facebook “addiction”. Psychol Rep (2014) 115:675–95. 10.2466/18.PR0.115c31z8 25489985

[70] Turel O Bechara A Social networking site use while driving: ADHD and the mediating roles of stress, self-esteem and craving. Front Psychol (2016) 7:455 10.3389/fpsyg.2016.00455 27065923 PMC4812103

[71] Turel O Bechara A Effects of motor impulsivity and sleep quality on swearing, interpersonally deviant and disadvantageous behaviors on online social networking sites. Person Individ Differ (2017) 108:91–7. 10.1016/j.paid.2016.12.005

[72] Feng Q Chen X Sun J Zhou Y Sun Y Ding W Voxel-level comparison of arterial spin-labeled perfusion magnetic resonance imaging in adolescents with internet gaming addiction. Behav Brain Func (2013) 9:33 10.1186/1744-9081-9-33 PMC3751515 23937918

[73] Hong S-B Zalesky A Cocchi L Fornito A Choi E-J Kim H-H Decreased functional brain connectivity in adolescents with internet addiction. PLoS One (2013) 8:e57831. 10.1371/journal.pone.0057831 23451272 PMC3581468

[74] Yuan K Yu D Cai C Feng D Li Y Bi Y Frontostriatal circuits, resting state functional connectivity and cognitive control in internet gaming disorder. Addict Biol (2017) 22:813–22. 10.1111/adb.12348 26769234

[75] Sun Y Ying H Seetohul RM Xuemei W Ya Z Qian L Brain fMRI study of crave induced by cue pictures in online game addicts (male adolescents). Behav Brain Res (2012) 233:563–76. 10.1016/j.bbr.2012.05.005 22684084

[76] Liu L Yip SW Zhang JT Wang LJ Shen ZJ Liu B Activation of the ventral and dorsal striatum during cue reactivity in Internet gaming disorder. Addict Biol (2017) 22:791–801. 10.1111/adb.12338 26732520 PMC5563850

[77] Hofmann W Friese M Wiers RW Impulsive versus reflective influences on health behavior: a theoretical framework and empirical review. Health Psychol Rev (2008) 2:111–37. 10.1080/17437190802617668

[78] Droutman V Read SJ Bechara A. Revisiting the role of the insula in addiction. Trends Cogn Sci (2015) 19:414–20. 10.1016/j.tics.2015.05.005 26066588 PMC4486609

[79] Zelazo PD Müller U Executive function in typical and atypical development. In: Goswami U, editor. Blackwell Handbook of Childhood Cognitive Development. Malden, MA: Blackwell Publishers Ltd (2002).

[80] Brand M Labudda K Markowitsch HJ. Neuropsychological correlates of decision-making in ambiguous and risky situations. Neural Netw (2006) 19:1266–76. 10.1016/j.neunet.2006.03.001 16942857

[81] Moreno-López L Stamatakis EA Fernández-Serrano MJ Gómez-Río M Rodríguez-Fernández A Pérez-García M Neural correlates of hot and cold executive functions in polysubstance addiction: association between neuropsychological performance and resting brain metabolism as measured by positron emission tomography. Psychiatry Res Neuroimag (2012) 203:214–21. 10.1016/j.pscychresns.2012.01.006 22959812

[82] Bechara A. The role of emotion in decision-making: evidence from neurological patients with orbitofrontal damage. Brain Cogn (2004) 55:30–40. 10.1016/j.bandc.2003.04.001 15134841

[83] Kerr A Zelazo PD Development of “hot” executive function: the children’s gambling task. Brain Cogn (2004) 55:148–57. 10.1016/S0278-2626(03)00275-6 15134849

[84] Sun D-L Chen Z-J Ma N Zhang X-C Fu X-M Zhang D-R. Decision-making and prepotent response inhibition functions in excessive internet users. CNS Spectr (2009) 14:75–81. 10.1017/S1092852900000225 19238122

[85] Bailey K West R Kuffel J. What would my avatar do? Gaming, pathology, and risky decision making. Front Psychol (2013) 4:409. 10.3389/fpsyg.2013.00609 24058356 PMC3767905

[86] Bechara A Damasio H Tranel D Damasio AR. The Iowa gambling task and the somatic marker hypothesis: some questions and answers. Trends Cogn Sci (2005) 9:159–62. 10.1016/j.tics.2005.02.002 15808493

[87] Qi X Yang Y Dai S Gao P Du X Zhang Y Effects of outcome on the covariance between risk level and brain activity in adolescents with internet gaming disorder. Neuroimage Clin (2016) 12:845–51. 10.1016/j.nicl.2016.10.024 27857886 PMC5103101

[88] Lin X Zhou H Dong G Du X. Impaired risk evaluation in people with Internet gaming disorder: fMRI evidence from a probability discounting task. Prog Neuropsychopharmacol Biol Psychiatry (2015) 56:142–8. 10.1016/j.pnpbp.2014.08.016 25218095

[89] Metcalf O Pammer K. Impulsivity and related neuropsychological features in regular and addictive first person shooter gaming. Cyberpsychol Behav Soc Netw (2014) 17:147–52. 10.1089/cyber.2013.0024 23971428

[90] Kim EJ Namkoong K Ku T Kim SJ. The relationship between online game addiction and aggression, self-control and narcissistic personality traits. Eur Psychiatry (2008) 23:212–8. 10.1016/j.eurpsy.2007.10.010 18166402

[91] Mehroof M Griffiths MD Online gaming addiction: the role of sensation seeking, self-control, neuroticism, aggression, state anxiety, and trait anxiety. Cyberpsychol Behav Soc Netw (2010) 13:313–6. 10.1089/cyber.2009.0229 20557251

[92] Wallenius M Punamäki R-L Digital game violence and direct aggression in adolescence: a longitudinal study of the roles of sex, age, and parent–child communication. J Appl Dev Psychol (2008) 29:286–94. 10.1016/j.appdev.2008.04.010

[93] Figueredo AJ Jacobs WJ Aggression, risk-taking, and alternative life history strategies: the behavioral ecology of social deviance. In: Frias-Armenta M Corral-Verdugo V, editors. Bio-Psycho-Social Perspectives on Interpersonal Violence. Nova Science Publishers, Inc (2011).

[94] Yuan K Cheng P Dong T Bi Y Xing L Yu D Cortical thickness abnormalities in late adolescence with online gaming addiction. PLoS One (2013) 8:e53055. 10.1371/journal.pone.0053055 23326379 PMC3541375

[95] Tian M Chen Q Zhang Y Du F Hou H Chao F PET imaging reveals brain functional changes in internet gaming disorder. Eur J Nucl Med Mol Imaging (2014) 41:1388–97. 10.1007/s00259-014-2708-8 24737115

[96] Takeuchi H Taki Y Hashizume H Asano K Asano M Sassa Y Impact of videogame play on the brain’s microstructural properties: cross-sectional and longitudinal analyses. Mol Psychiatry (2016) 21:1781–9. 10.1038/mp.2015.193 26728566 PMC5116480

[97] Blair R. The roles of orbital frontal cortex in the modulation of antisocial behavior. Brain Cogn (2004) 55:198–208. 10.1016/S0278-2626(03)00276-8 15134853

[98] Rolls ET Grabenhorst F. The orbitofrontal cortex and beyond: from affect to decision-making. Prog Neurobiol (2008) 86:216–44. 10.1016/j.pneurobio.2008.09.001 18824074

[99] Greitemeyer T Mügge DO. Video games do affect social outcomes: a meta-analytic review of the effects of violent and prosocial video game play. Pers Soc Psychol Bull (2014) 40:578–89. 10.1177/0146167213520459 24458215

[100] Hare TA Camerer CF Rangel A. Self-control in decision-making involves modulation of the vmPFC valuation system. Science (2009) 324:646–8. 10.1126/science.1168450 19407204

[101] Ko C-H Hsieh T-J Chen C-Y Yen C-F Chen C-S Yen J-Y Altered brain activation during response inhibition and error processing in subjects with Internet gaming disorder: a functional magnetic imaging study. Eur Arch Psychiatry Clin Neurosci (2014) 264:661–72. 10.1007/s00406-013-0483-3 24469099

[102] Houben K Wiers RW. Implicitly positive about alcohol? Implicit positive associations predict drinking behavior. Addict Behav (2008) 33:979–86. 10.1016/j.addbeh.2008.03.002 18434034

[103] Menon V Adleman NE White CD Glover GH Reiss AL Error-related brain activation during a Go/NoGo response inhibition task. Hum Brain Mapp (2001) 12:131–43. 10.1002/1097-0193(200103)12:3<131::AID-HBM1010>3.0.CO;2-C 11170305 PMC6872006

[104] Littel M Berg I Luijten M Rooij AJ Keemink L Franken IH Error processing and response inhibition in excessive computer game players: an event-related potential study. Addict Biol (2012) 17:934–47. 10.1111/j.1369-1600.2012.00467.x 22734609

[105] Ding W-N Sun J-H Sun Y-W Chen X Zhou Y Zhuang Z-G Trait impulsivity and impaired prefrontal impulse inhibition function in adolescents with internet gaming addiction revealed by a Go/No-Go fMRI study. Behav Brain Func (2014) 10:20 10.1186/1744-9081-10-20 PMC4050412 24885073

[106] Chen CY Huang MF Yen JY Chen CS Liu GC Yen CF Brain correlates of response inhibition in Internet gaming disorder. Psychiatry Clin Neurosci (2015) 69:201–9. 10.1111/pcn.12224 25047685

[107] Kim M Lee TH Choi J-S Kwak YB Hwang WJ Kim T Neurophysiological correlates of altered response inhibition in internet gaming disorder and obsessive-compulsive disorder: perspectives from impulsivity and compulsivity. Sci Rep (2017) 7:41742. 10.1038/srep41742 28134318 PMC5278399

[108] Irvine MA Worbe Y Bolton S Harrison NA Bullmore ET Voon V. Impaired decisional impulsivity in pathological videogamers. PLoS One (2013) 8:e75914. 10.1371/journal.pone.0075914 24146789 PMC3797823

[109] Choi S-W Kim H Kim G-Y Jeon Y Park S Lee J-Y Similarities and differences among Internet gaming disorder, gambling disorder and alcohol use disorder: a focus on impulsivity and compulsivity. J Behav Addict (2014) 3:246–53. 10.1556/JBA.3.2014.4.6 25592310 PMC4291830

[110] Tanaka S Ikeda H Kasahara K Kato R Tsubomi H Sugawara SK Larger right posterior parietal volume in action video game experts: a behavioral and voxel-based morphometry (VBM) study. PLoS One (2013) 8:e66998. 10.1371/journal.pone.0066998 23776706 PMC3679077

[111] Kühn S Gallinat J. Amount of lifetime video gaming is positively associated with entorhinal, hippocampal and occipital volume. Mol Psychiatry (2014) 19:842. 10.1038/mp.2013.100 23958958

[112] Jin C Zhang T Cai C Bi Y Li Y Yu D Abnormal prefrontal cortex resting state functional connectivity and severity of internet gaming disorder. Brain Imaging Behav (2016) 10:719–29. 10.1007/s11682-015-9439-8 26311395

[113] Liu G-C Yen J-Y Chen C-Y Yen C-F Chen C-S Lin W-C Brain activation for response inhibition under gaming cue distraction in internet gaming disorder. Kaohsiung J Med Sci (2014) 30:43–51. 10.1016/j.kjms.2013.08.005 24388058 PMC11916293

[114] Han DH Hwang JW Renshaw PF. Bupropion sustained release treatment decreases craving for video games and cue-induced brain activity in patients with Internet video game addiction. Exp Clin Psychopharmacol (2010) 18:297. 10.1037/a0020023 20695685

[115] Friedman NP Miyake A The relations among inhibition and interference control functions: a latent-variable analysis. J Exp Psychol (2004) 133:101 10.1037/0096-3445.133.1.101 14979754

[116] Robinson TE Berridge KC. The neural basis of drug craving: an incentive-sensitization theory of addiction. Brain Res Rev (1993) 18:247–91. 10.1016/0165-0173(93)90013-P 8401595

[117] Goldstein RZ Craig AD Bechara A Garavan H Childress AR Paulus MP The neurocircuitry of impaired insight in drug addiction. Trends Cogn Sci (2009) 13:372–80. 10.1016/j.tics.2009.06.004 19716751 PMC2844118

[118] Naqvi NH Bechara A. The hidden island of addiction: the insula. Trends Neurosci (2009) 32:56–67. 10.1016/j.tins.2008.09.009 18986715 PMC3698860

[119] Goldstein RZ Volkow ND. Dysfunction of the prefrontal cortex in addiction: neuroimaging findings and clinical implications. Nat Rev Neurosci (2011) 12:652–69. 10.1038/nrn3119 22011681 PMC3462342

[120] Craig AD How do you feel – now? The anterior insula and human awareness. Nat Rev Neurosci (2009) 10:59–70. 10.1038/nrn2555 19096369

[121] Contreras M Ceric F Torrealba F. Inactivation of the interoceptive insula disrupts drug craving and malaise induced by lithium. Science (2007) 318:655–8. 10.2307/20051463 17962567

[122] Garavan H. Insula and drug cravings. Brain Struct Funct (2010) 214:593–601. 10.1007/s00429-010-0259-8 20512373

[123] Delgado MR Nystrom LE Fissell C Noll D Fiez JA. Tracking the hemodynamic responses to reward and punishment in the striatum. J Neurophysiol (2000) 84:3072–7. 11110834 10.1152/jn.2000.84.6.3072

[124] Samanez-Larkin GR Hollon NG Carstensen LL Knutson B. Individual differences in insular sensitivity during loss anticipation predict avoidance learning. Psychol Sci (2008) 19:320–3. 10.1111/j.1467-9280.2008.02087.x 18399882 PMC2365707

[125] Morales AM Ghahremani D Kohno M Hellemann GS London ED. Cigarette exposure, dependence, and craving are related to insula thickness in young adult smokers. Neuropsychopharmacology (2014) 39:1816. 10.1038/npp.2014.48 24584328 PMC4059909

[126] Naqvi NH Rudrauf D Damasio H Bechara A. Damage to the insula disrupts addiction to cigarette smoking. Science (2007) 315:531–4. 10.1126/science.1135926 17255515 PMC3698854

[127] Paulus MP Frank LR. Ventromedial prefrontal cortex activation is critical for preference judgments. Neuroreport (2003) 14:1311. 10.1097/01.wnr.0000078543.07662.02 12876463

[128] Chen C-Y Yen J-Y Wang P-W Liu G-C Yen C-F Ko C-H. Altered functional connectivity of the insula and nucleus accumbens in Internet gaming disorder: a resting state fMRI study. Eur Addict Res (2016) 22:192–200. 10.1159/000440716 26863028

[129] Zhang Y Mei W Zhang JX Wu Q Zhang W. Decreased functional connectivity of insula-based network in young adults with internet gaming disorder. Exp Brain Res (2016) 234:2553–60. 10.1007/s00221-016-4659-8 27119360

[130] Naqvi NH Bechara A. The insula and drug addiction: an interoceptive view of pleasure, urges, and decision-making. Brain Struct Funct (2010) 214:435–50. 10.1007/s00429-010-0268-7 20512364 PMC3698865

[131] Verdejo-García A Lubman DI Schwerk A Roffel K Vilar-López R MacKenzie T Effect of craving induction on inhibitory control in opiate dependence. Psychopharmacology (2012) 219:519–26. 10.1007/s00213-011-2512-0 21952672

[132] Gauggel S Heusinger A Forkmann T Boecker M Lindenmeyer J Miles Cox W Effects of alcohol cue exposure on response inhibition in detoxified alcohol-dependent patients. Alcoholism (2010) 34:1584–9. 10.1111/j.1530-0277.2010.01243.x 20586755

[133] Zhou Y Lin F-C Du Y-S Zhao Z-M Xu J-R Lei H Gray matter abnormalities in Internet addiction: a voxel-based morphometry study. Eur J Radiol (2011) 79:92–5. 10.1016/j.ejrad.2009.10.025 19926237

[134] Goldstein RZ Volkow ND. Drug addiction and its underlying neurobiological basis: neuroimaging evidence for the involvement of the frontal cortex. Am J Psychiatry (2002) 159:1642–52. 10.1176/appi.ajp.159.10.1642 12359667 PMC1201373

[135] Gentile DA Choo H Liau A Sim T Li D Fung D Pathological video game use among youths: a two-year longitudinal study. Pediatrics (2011) 127(2):e319–29. 10.1542/peds.2010-1353 21242221

[136] Avery JA Drevets WC Moseman SE Bodurka J Barcalow JC Simmons WK. Major depressive disorder is associated with abnormal interoceptive activity and functional connectivity in the insula. Biol Psychiatry (2014) 76:258–66. 10.1016/j.biopsych.2013.11.027 24387823 PMC4048794

[137] Brevers D Cleeremans A Bechara A Greisen M Kornreich C Verbanck P Impaired self-awareness in pathological gamblers. J Gambl Stud (2013) 29:119–29. 10.1007/s10899-012-9292-2 22273773

[138] Nelson TO Metamemory: a theoretical framework and new findings. Psychol Learn Motiv (1990) 26:125–73. 10.1016/S0079-7421(08)60053-5

[139] Pujol J Fenoll R Forns J Harrison BJ Martinez-Vilavella G Macia D Video gaming in school children: how much is enough? Ann Neurol (2016) 80:424–33. 10.1002/ana.24745 27463843

[140] Davis RA A cognitive–behavioral model of pathological Internet use. Comput Human Behav (2001) 17:187–95. 10.1016/S0747-5632(00)00041-8

[141] Dong G Potenza MN. A cognitive-behavioral model of Internet gaming disorder: theoretical underpinnings and clinical implications. J Psychiatr Res (2014) 58:7–11. 10.1016/j.jpsychires.2014.07.005 25062755 PMC4448942

[142] Han DH Lee YS Na C Ahn JY Chung US Daniels MA The effect of methylphenidate on Internet video game play in children with attention-deficit/hyperactivity disorder. Compr Psychiatry (2009) 50:251–6. 10.1016/j.comppsych.2008.08.011 19374970

[143] Young KS. Cognitive behavior therapy with Internet addicts: treatment outcomes and implications. Cyberpsychol Behav (2007) 10:671–9. 10.1089/cpb.2007.9971 17927535

